# Comparative impact of different weight loss strategies on obstructive sleep apnea: an integrated review of surgical, pharmacological, and lifestyle interventions

**DOI:** 10.3389/fneur.2025.1719923

**Published:** 2025-12-15

**Authors:** Guo-qiang Song, Guo-qiang Hu

**Affiliations:** Department of Pulmonary, Changxing County Hospital of Traditional Chinese Medicine, Huzhou, China

**Keywords:** obstructive sleep apnea, weight reduction, bariatric surgery, pharmacological therapy, weight management, GLP-1 receptor agonists

## Abstract

Obesity is recognized as one of the most significant risk factors for obstructive sleep apnea (OSA), and weight reduction remains an effective strategy for improving OSA symptoms. With the ongoing evolution of bariatric surgery, pharmacological therapies, and conventional weight management approaches such as dietary modification and exercise, there is growing interest in understanding the differential efficacy and mechanisms of these interventions for OSA management. This review systematically examines the impacts of surgical procedures (including bariatric surgeries like gastric sleeve), pharmacological treatments (such as GLP-1 receptor agonists, orlistat, and phentermine/topiramate), and lifestyle-based weight management on OSA outcomes. It analyzes the comparative effectiveness, underlying mechanisms, indications, and limitations of each approach, integrating insights from the latest clinical studies. Additionally, this review discusses the challenges and unresolved issues in the field, such as patient selection, long-term adherence, and the interplay between weight loss and OSA pathophysiology. The purpose of this article is to provide a comprehensive synthesis of current evidence, highlight gaps in knowledge, and outline future directions for integrating weight management strategies into the holistic care of patients with OSA.

## 1 Introduction

Obstructive sleep apnea (OSA) is a highly prevalent sleep disorder characterized by recurrent episodes of upper airway obstruction during sleep, leading to intermittent hypoxia, sleep fragmentation, and significant physiological stress. The global burden of OSA is substantial, with estimates suggesting that nearly one billion people are affected worldwide, although many cases remain undiagnosed (1, 2). Epidemiological studies consistently demonstrate a strong association between obesity and OSA, with the prevalence of OSA markedly increased in individuals with elevated body mass index (BMI) (3–5). The full etiology of OSA is multifactorial, involving anatomical, neuromuscular, and genetic factors, with specific risk factors including craniofacial anomalies; disputable associations, such as the coincidence of malocclusions (e.g., Class II or III) with OSA, may involve airway narrowing, though causality remains unclear and requires further polysomnography and interdisciplinary research (6). Obesity contributes to OSA pathogenesis through several mechanisms, including increased fat deposition in the upper airway, reduced airway caliber, and altered neuromuscular control, all of which predispose to airway collapse during sleep (7, 8). The bidirectional relationship between obesity and OSA is further evidenced by the fact that OSA can exacerbate metabolic dysfunction, promote weight gain through disrupted sleep and hormonal changes, and increase the risk of developing obesity-related comorbidities (9, 10). Importantly, OSA is not limited to adults; it is also prevalent among children and adolescents with obesity, where it can contribute to a range of adverse health outcomes (11–13). The high prevalence of OSA in special populations, such as pregnant women with class III obesity and patients with metabolic syndrome, underscores the need for heightened clinical vigilance and targeted screening strategies (14–16). Epidemiologically, OSA prevalence is approximately 2–3 times higher in men than women, rises with age (peaking in midlife), and is linked to congenital diseases like Treacher Collins Syndrome (~1:50,000 live births), where craniofacial malformations such as mandibular hypoplasia contribute to OSA in up to 95% of cases, with progressive severity over time (17).

The clinical implications of OSA extend far beyond impaired sleep quality. Untreated OSA is associated with a spectrum of adverse health outcomes, including increased risk of cardiovascular diseases (such as hypertension, arrhythmias, coronary artery disease, heart failure, and stroke), metabolic syndrome, type 2 diabetes, chronic kidney disease, and neurocognitive impairments (1, 18–20). The pathophysiological mechanisms underlying these associations are complex and multifactorial, involving intermittent hypoxemia, oxidative stress, systemic inflammation, sympathetic nervous system activation, and endothelial dysfunction (21–24). OSA is also linked to increased morbidity and mortality, reduced quality of life, impaired workplace productivity, and elevated risk of accidents (18, 25, 26). The burden of OSA-related comorbidities is particularly pronounced in individuals with obesity, where the coexistence of metabolic, cardiovascular, and sleep disorders creates a vicious cycle that amplifies health risks and complicates management (27, 28). Beyond the listed outcomes, OSA influences daily life by elevating cardiovascular risks, including arrhythmias and sudden cardiac events, particularly in obese individuals where obesity-related factors like intermittent hypoxia and disrupted circadian rhythms (e.g., reduced NPAS2 protein levels) intensify metabolic disturbances and cardiovascular burden (29). Early recognition, accurate diagnosis—often via polysomnography—and comprehensive management are essential to mitigate these risks and improve patient outcomes (11, 30).

Weight loss is universally recognized as a cornerstone non-surgical intervention for the management of OSA, particularly in patients with overweight or obesity (31, 32). Even modest reductions in body weight can lead to significant improvements in OSA severity, as measured by reductions in the apnea-hypopnea index (AHI), and can ameliorate associated cardiometabolic risk factors (33, 34). However, the effectiveness of different weight loss modalities—including lifestyle interventions, pharmacotherapy, and bariatric surgery—varies considerably, and the durability of weight loss and OSA remission remains a clinical challenge (35–37). Lifestyle modifications involving dietary changes, increased physical activity, and behavioral therapy are first-line strategies but often yield limited long-term success in achieving and maintaining clinically meaningful weight loss (13, 38). Pharmacological therapies, particularly newer incretin-based agents, have shown promise in facilitating sustained weight loss and improving OSA, though issues of cost and long-term safety persist (26, 39, 40). Per the American Academy of Sleep Medicine (AASM) 2017 clinical practice guideline on diagnostic testing for adult OSA, diagnosis typically involves polysomnography or home sleep apnea testing, with treatment guidelines emphasizing non-invasive therapies like continuous positive airway pressure (CPAP) as first-line and surgical interventions as a last resort after failures of conservative approaches (41). Bariatric surgery remains the most effective intervention for substantial and sustained weight loss, with robust evidence supporting its role in improving or resolving OSA and related comorbidities, especially in patients with severe obesity (35, 42, 43). Nevertheless, the selection of optimal weight loss strategies must be individualized, taking into account patient characteristics, comorbidity profiles, and the potential risks and benefits of each intervention (31, 44).

With ongoing advancements in surgical techniques, pharmacological agents, and comprehensive weight management approaches, there is growing clinical interest in the comparative efficacy, indications, and safety profiles of these interventions for OSA (35, 39, 45). Recent randomized controlled trials and meta-analyses have provided valuable insights into the proportional relationship between the degree of weight loss and improvement in OSA severity, as well as the impact of multidisciplinary interventions on cardiometabolic health and quality of life (34, 45, 46). However, challenges remain in identifying patients most likely to benefit from specific interventions, optimizing long-term adherence, and addressing barriers to sustained weight loss and OSA remission (36, 37). Furthermore, the heterogeneity of OSA phenotypes, the presence of non-anatomical contributors to airway obstruction, and the influence of sex, age, and comorbid conditions necessitate a personalized, evidence-based approach to therapy (8, 16, 44). This review aims to systematically synthesize current evidence on the effects and mechanisms of different weight loss strategies—including surgical, pharmacological, and lifestyle interventions—on OSA, providing clinicians with a comprehensive framework to guide individualized management and improve outcomes for patients with this multifaceted disorder.

## 2 Main text

### 2.1 The efficacy and mechanisms of bariatric surgery on OSA

#### 2.1.1 Common types of bariatric surgery and indications

Bariatric surgery encompasses a variety of surgical procedures designed to induce significant and sustained weight loss, particularly in individuals with severe obesity and related metabolic complications such as OSA. The two most commonly performed bariatric procedures worldwide are sleeve gastrectomy (SG) and Roux-en-Y gastric bypass (RYGB), both of which have demonstrated robust efficacy in reducing body weight and ameliorating comorbid conditions (47, 48). SG involves the resection of a large portion of the stomach, resulting in a tubular gastric remnant that restricts food intake and alters gut hormone secretion. RYGB, on the other hand, creates a small gastric pouch and reroutes the small intestine, combining restrictive and malabsorptive mechanisms to promote weight loss and metabolic improvements (48, 49). Indications for bariatric surgery have evolved over time; current international guidelines generally recommend these procedures for individuals with a BMI ≥ 40 kg/m^2^ or those with BMI ≥ 35 kg/m^2^ who have significant obesity-related comorbidities, such as type 2 diabetes, hypertension, or OSA (47, 50). Recent consensus statements and updates from surgical societies now also consider patients with BMI 30–34.9 kg/m^2^ eligible if they have poorly controlled metabolic diseases, reflecting the growing recognition of the metabolic benefits of surgery beyond weight loss alone (50, 51). The choice of surgical procedure is influenced by patient-specific factors, including the presence and severity of comorbidities, anatomical considerations, and patient preference. Both SG and RYGB are typically performed laparoscopically, which minimizes operative risk and recovery time (48, 52). Importantly, for patients with BMI ≥ 35 kg/m^2^ and OSA, bariatric surgery is strongly indicated due to its superior efficacy in reducing body weight and improving OSA symptoms compared to non-surgical interventions (47, 49). In summary, SG and RYGB are the principal bariatric procedures, with clear indications and procedural guidelines, particularly for patients with severe obesity and associated metabolic diseases such as OSA.

#### 2.1.2 Surgical weight loss and improvement in OSA severity

Multiple studies have demonstrated that bariatric surgery leads to significant reductions in the apnea-hypopnea index (AHI), a key metric for assessing the severity of OSA, and in some cases, complete resolution of OSA is achieved. A comprehensive systematic review and meta-analysis encompassing 2,310 patients found that bariatric surgery resulted in a weighted mean difference reduction in AHI of −19.3 events/h, and the rate of OSA remission post-surgery was 65% (53). This improvement is not only statistically significant but also clinically meaningful, as lower AHI values are associated with reduced morbidity and improved quality of life. Further supporting these findings, a large narrative review based on the Scandinavian Obesity Surgery Registry reported significant long-term improvements in sleep apnea following gastric bypass, with a corresponding decrease in related comorbidities (54). The magnitude of weight loss achieved through bariatric procedures is positively correlated with the degree of OSA improvement, as demonstrated by a recent meta-analysis quantifying the relationship between weight reduction and AHI change: a 20% reduction in BMI was associated with a 57% decrease in AHI, though further reductions in BMI beyond this threshold produced diminishing returns in AHI improvement (55). Notably, both metabolically healthy and unhealthy obese patients benefit similarly from laparoscopic sleeve gastrectomy in terms of OSA remission, indicating that the metabolic profile does not substantially alter the beneficial effects of surgical weight loss on OSA (56). Individual cohort studies corroborate these findings, showing that after bariatric surgery, not only is there a significant drop in AHI, but also a reduction in the prevalence and severity of OSA, with some patients achieving complete remission (AHI < 5 events/h) (57). However, it is important to note that while a majority of patients experience significant improvement, complete resolution of OSA is achieved in less than half of cases, suggesting that factors beyond obesity—such as craniofacial anatomy and upper airway characteristics—also play a role in OSA persistence (58, 59). Overall, the evidence robustly supports bariatric surgery as an effective intervention for reducing OSA severity, with the extent of weight loss being a key determinant of the degree of improvement.

#### 2.1.3 Mechanistic analysis of surgical weight loss

Bariatric surgery improves OSA primarily through mechanisms that address the anatomical and physiological contributors to airway obstruction in obese individuals. One of the key pathways is the reduction of pharyngeal fat deposition, which leads to enlargement of the upper airway and a decrease in its collapsibility during sleep. Imaging studies have demonstrated that weight loss after bariatric surgery is associated with significant reductions in tongue fat and lateral pharyngeal wall volumes, both of which are strongly correlated with improvements in AHI and overall OSA severity (60). Acoustic pharyngometry and polysomnographic assessments confirm that post-surgical patients exhibit increased pharyngeal cross-sectional area, especially at the oropharyngeal junction and glottal regions, which directly enhances airway patency and reduces obstructive events (61). Additionally, weight loss achieved through surgery results in decreased neck circumference and waist circumference, further alleviating the mechanical load on respiratory structures (62).

Beyond anatomical changes, bariatric surgery also induces favorable metabolic and inflammatory shifts that contribute to OSA improvement. Postoperative reductions in insulin resistance and systemic inflammation have been observed, as evidenced by associations between lower levels of high-sensitivity C-reactive protein, triglycerides, and HbA1c and reduced OSA risk scores, although direct causal reductions in these markers with OSA symptom improvement require further study (63). These metabolic enhancements may reduce the propensity for upper airway edema and neuromuscular dysfunction, thereby supporting airway stability during sleep. Furthermore, the improvement in pulmonary function parameters—such as forced vital capacity and oxygen saturation—following surgical weight loss underscores the role of reduced respiratory load and enhanced ventilatory efficiency in OSA amelioration (64). Collectively, these findings highlight that bariatric surgery addresses both the structural and functional determinants of OSA, offering a multifaceted therapeutic benefit that extends beyond simple weight reduction. However, it is important to note that while a majority of patients experience significant OSA improvement or remission after surgery, complete resolution is not universal, suggesting that non-obesity-related anatomical and physiological factors may also play a role in persistent OSA (53).

#### 2.1.4 Limitations and complications of bariatric surgery

Despite its proven efficacy in achieving significant and sustained weight loss and improving obesity-related comorbidities, bariatric surgery is associated with several important limitations and potential complications that must be carefully considered, particularly in specific patient populations. Surgical risks inherent to bariatric procedures include perioperative complications and mortality, with complication rates varying based on surgical technique, patient comorbidities, and surgical expertise (65). Nutritional deficiencies are a well-documented concern following bariatric surgery, especially with malabsorptive procedures like RYGB and biliopancreatic diversion. Patients are at increased risk for deficiencies in protein, iron, calcium, vitamin B12, folate, and other micronutrients, which can manifest as anemia, osteoporosis, neuropathies, and other systemic complications (66–68).

Moreover, while bariatric surgery typically results in substantial weight loss, there is a recognized risk of weight regain or insufficient weight loss, leading to the need for revisional surgeries. Revisional procedures, performed due to complications or failure of the primary surgery, carry higher risks of morbidity and technical complexity (68). In addition, certain complications such as gastrointestinal ulcers, strictures, fistulas, gallstone disease, kidney stones, and rare but severe events like liver failure, splenic abscess, or rhabdomyolysis have been reported after bariatric surgery (69–71). The risk of complications is further amplified in elderly patients or those with significant comorbidities such as advanced liver disease, cardiovascular disease, or poor functional status. Although recent evidence suggests that bariatric surgery can be performed safely in selected elderly patients, these individuals still experience higher rates of early postoperative complications, and careful patient selection and perioperative management are essential (72, 73). Patients with advanced liver fibrosis or cirrhosis require thorough preoperative assessment, as the risk of hepatic decompensation, longer hospital stays, and increased perioperative morbidity is higher, though studies show that with proper selection, bariatric surgery can be performed with acceptable safety profiles (74–76).

Finally, psychosocial and behavioral considerations are critical, as postoperative mental health issues—including depression, substance use disorders, and even increased risk of suicide—have been documented, underscoring the need for long-term multidisciplinary follow-up (77, 78). In summary, while bariatric surgery is a powerful tool against severe obesity and related conditions, its limitations—including surgical risks, nutritional complications, risk of recurrence, and special considerations in high-risk populations—necessitate individualized risk–benefit assessment, patient education, and comprehensive long-term care.

### 2.2 The effects of conventional weight management programs on OSA

#### 2.2.1 Effects of dietary control and exercise interventions

Dietary control and exercise interventions remain foundational strategies in weight management for patients with OSA, with compelling evidence supporting their role in reducing OSA severity and improving metabolic health. Energy-restricted diets, such as low-calorie or low-carbohydrate regimens, have demonstrated efficacy in promoting weight loss and, consequently, alleviating OSA symptoms. For example, a randomized clinical trial evaluating the Mediterranean diet combined with lifestyle intervention found that, when added to standard care (continuous positive airway pressure, CPAP, and brief healthy lifestyle advice), the intervention led to a significantly greater reduction in AHI compared to standard care alone, independent of weight loss, suggesting benefits beyond mere caloric restriction (79). Similarly, alternate day fasting combined with a low-carbohydrate diet resulted in sustained weight and fat mass reduction, although it did not significantly impact sleep quality, insomnia severity, or OSA risk in adults with obesity, highlighting that not all dietary approaches uniformly affect OSA outcomes (80).

Exercise, encompassing both aerobic and resistance training, further augments the benefits of dietary interventions. Structured lifestyle modification programs that integrate individualized plans for diet and physical activity have been shown to significantly improve cardiorespiratory capacity and reduce body weight and BMI, with corresponding improvements in health-related quality of life among participants with class II and III obesity (81). In obese adolescents, multidisciplinary interventions combining diet and exercise led to improvements in cardiometabolic risk factors and, notably, normalization of sleep-disordered breathing in a significant proportion of participants (82). Additionally, studies indicate that adding regular exercise—such as daily free walking—to a low-calorie diet yields greater improvements in daytime sleepiness, anthropometric measures, and OSA severity than dietary intervention alone (83).

Aerobic exercise and resistance training may also directly enhance upper airway muscle function, potentially reducing airway collapsibility during sleep and thereby improving OSA independent of weight loss. Moreover, lifestyle interventions that encompass healthy diet and regular physical activity are associated with a lower risk of insomnia, excessive daytime sleepiness, and OSA in large population studies (84). However, the magnitude of benefit can vary, and long-term adherence remains a challenge. Qualitative research underscores the importance of personalized, psychologically informed, and technologically integrated programs to support sustainable weight loss and lifestyle change in the sleep medicine population (85). In summary, energy-restricted diets and structured exercise regimens are effective for weight reduction and can significantly improve OSA severity and upper airway function, especially when delivered as part of comprehensive, individualized lifestyle interventions.

#### 2.2.2 Behavioral interventions and multidisciplinary weight management

Behavioral interventions, particularly cognitive-behavioral therapy (CBT), combined with robust family and social support, play a pivotal role in achieving and maintaining long-term weight control and alleviating OSA. The efficacy of behavioral and lifestyle interventions as first-line strategies for obesity management is well established (86, 87). Cognitive-behavioral-psychological interventions, when integrated with family-based weight management, have shown superior outcomes compared to conventional management alone. In a clinical study of obese OSA patients, those receiving uvulopalatopharyngoplasty (UPPP) in conjunction with cognitive-behavioral and family weight management exhibited greater reductions in BMI, neck and waist circumference, and more substantial improvements in AHI and oxygen saturation parameters than those receiving standard care, underscoring the synergistic effect of behavioral strategies and family engagement (88).

Multidisciplinary approaches, which often include nutrition therapy, physical activity, behavioral counseling, and medical interventions, are increasingly recognized as essential for effective and sustainable weight reduction (89, 90). These comprehensive programs not only address dietary and activity modifications but also leverage the motivational and practical support provided by family and social networks. For example, family-based interventions have been shown to enhance adherence to nutritional and physical activity recommendations in individuals with obesity and comorbid conditions such as type 2 diabetes, further supporting the importance of social support in facilitating behavior change and health improvement (91). Moreover, healthy lifestyle behaviors—including high-quality diets, regular physical activity, and weight maintenance—are independently associated with reduced OSA risk, with evidence indicating that BMI mediates much of this protective effect (92). Importantly, even after accounting for BMI, the direct benefits of healthy lifestyle adherence on OSA remain significant, highlighting the multifaceted impact of behavioral interventions.

In summary, behavioral interventions, especially when embedded within a multidisciplinary framework and supported by family and social networks, are important to the long-term control of body weight and the mitigation of OSA severity. These approaches foster sustainable health behaviors, improve treatment adherence, and enhance clinical outcomes, reinforcing the need for comprehensive, patient-centered strategies in the management of obesity-related OSA.

#### 2.2.3 Evidence for the efficacy of weight management in OSA

Clinical trials and systematic reviews consistently affirm that weight management interventions can significantly improve OSA outcomes, particularly by reducing the AHI, alleviating daytime sleepiness, and enhancing quality of life. Multidisciplinary weight loss programs, combining dietary modifications, physical activity, and behavioral therapy, have demonstrated notable reductions in OSA severity and normalization of OSA in a substantial proportion of youth and adults with obesity (38). In adults, structured diet management plans, especially when delivered as educational programs, have been shown to decrease AHI and improve nocturnal oxygenation and subjective sleep quality, even when used alongside CPAP therapy (93). The MIMOSA randomized clinical trial further highlighted that a Mediterranean diet and lifestyle intervention on top of standard care led to greater improvements in AHI and OSA symptoms than standard care alone, regardless of CPAP use or the degree of weight loss achieved, suggesting additional benefits from lifestyle modification beyond weight reduction per se (79). In pediatric populations, multidisciplinary interventions led to significant improvements in OSA severity and sleep duration (38), though recurrence rates remain high in obese children after adenotonsillectomy, emphasizing the need for sustained weight management and multidisciplinary approaches (94). Evidence from adult studies also shows a proportional relationship between the percentage of weight loss and reduction in AHI, with meta-regression analyses indicating that every 1% loss in body weight can yield a measurable improvement in OSA severity (45). However, the effectiveness of weight management is closely tied to patient adherence; long-term maintenance of weight loss is challenging, and lapses in adherence can attenuate the therapeutic benefits (37). In summary, while conventional weight management is a foundational and evidence-based approach for reducing OSA severity and improving related symptoms, its overall efficacy in practice is often moderated by patient adherence and the ability to maintain long-term lifestyle changes.

#### 2.2.4 Limitations and challenges

Despite the well-established benefits of weight loss in improving OSA, several significant limitations and challenges persist across surgical, pharmacological, and lifestyle-based interventions. One of the most pressing issues is the high rate of weight regain following initial weight loss, which undermines the long-term efficacy of these treatments. Studies have shown that while bariatric surgery and intensive lifestyle interventions can result in substantial weight loss and OSA improvement, a considerable proportion of patients experience weight recidivism over time, leading to the partial or full return of OSA symptoms (33, 95). Long-term adherence to lifestyle modifications, such as dietary changes and regular physical activity, is notoriously poor, with most patients struggling to maintain clinically significant weight loss without continued, structured support (13, 36). Pharmacological treatments, including GLP-1 receptor agonists and other anti-obesity medications, offer promise for sustained weight loss and OSA improvement, but real-world data indicate that their effectiveness is often limited by side effects, high cost, and suboptimal long-term adherence (39, 96). Furthermore, these medications may not be accessible to all patients due to financial or healthcare system barriers, and their discontinuation frequently leads to weight regain and OSA recurrence.

Another challenge is the limited efficacy of weight loss interventions in individuals with severe or morbid obesity. Evidence suggests that patients with higher baseline BMI or more severe OSA are less likely to achieve complete remission of OSA, even after substantial weight reduction, whether through surgery, medication, or lifestyle changes (42, 97). This limitation is particularly evident in adolescents and adults with extreme obesity, where the anatomical and physiological changes contributing to OSA may not be fully reversed by weight loss alone (42, 98). Additionally, certain patient subgroups, such as those with underlying genetic syndromes or complex comorbidities, may derive less benefit from standard weight loss approaches and require more individualized or multidisciplinary care (99, 100). Collectively, these challenges highlight the need for ongoing research into strategies that enhance long-term adherence, address weight regain, and improve outcomes for patients with severe obesity, as well as the importance of integrating weight management with other therapeutic modalities for optimal OSA control.

### 2.3 The impact of pharmacological weight loss therapy on OSA

#### 2.3.1 Glucagon-like peptide-1 receptor agonists (GLP-1 RAs, e.g., liraglutide, semaglutide)

Clinical research increasingly supports the efficacy of GLP-1 RAs, such as liraglutide and semaglutide, in achieving significant weight reduction and improving OSA symptoms in certain patient populations. Multiple meta-analyses and systematic reviews have demonstrated that GLP-1 RAs can lead to substantial decreases in body weight and BMI, which are closely linked to reductions in OSA severity, as measured by the AHI (101–103). For example, pooled data from randomized controlled trials indicate that GLP-1 RAs can reduce AHI by approximately 5–16 events per hour, with greater improvements observed in individuals with higher baseline obesity (101, 102). The weight loss achieved with these agents is clinically meaningful, with semaglutide and liraglutide consistently outperforming placebo and, in some studies, other anti-obesity medications (104, 105). The mechanisms underlying these benefits are multifactorial: GLP-1 RAs suppress appetite via central nervous system pathways, leading to reduced caloric intake, and also promote satiety and delay gastric emptying (106, 107). Additionally, emerging evidence suggests that these agents may exert anti-inflammatory effects and improve metabolic parameters such as glycemic control, blood pressure, and lipid profiles, all of which can further contribute to ameliorating OSA pathophysiology (108–110). Some studies also highlight the potential for GLP-1 RAs to reduce upper airway fat deposition and systemic inflammation, thereby directly targeting key contributors to OSA beyond weight loss alone (108, 111). However, while the overall safety profile of GLP-1 RAs is favorable, with gastrointestinal side effects being the most common, rare but serious adverse events such as delayed gastric emptying and psychiatric effects have been reported, necessitating careful patient selection and monitoring (112, 113). Dosage and formulation have been investigated and show differences; injectable GLP-1 agonists (e.g., semaglutide) typically yield superior weight loss efficacy compared to oral forms in obesity studies, attributed to better bioavailability and adherence, though OSA-specific comparisons are scarce (114, 115). This distinction is important, as it could amplify AHI reductions through enhanced weight loss, highlighting the need for targeted OSA research on long-term effects. In summary, GLP-1 RAs represent a promising pharmacological approach for obese patients with OSA, offering both direct and indirect mechanisms for symptom improvement, though further large-scale, long-term studies are warranted to optimize their use and clarify their role in OSA management (101, 102, 108).

#### 2.3.2 Orlistat and other traditional anti-obesity medications

Orlistat, a gastrointestinal lipase inhibitor, exerts its weight-reducing effects primarily by blocking the absorption of dietary fats, thereby promoting caloric deficit and subsequent weight loss. Its mechanism is distinct from centrally acting appetite suppressants, as it acts peripherally within the gastrointestinal tract. Clinical guidelines recommend pharmacotherapy, including orlistat, as an adjunct to lifestyle modifications for patients with obesity (BMI ≥ 30 kg/m^2^ or ≥27 kg/m^2^ with comorbidities such as OSA) when non-pharmacological interventions are insufficient (116). Evidence suggests that orlistat can modestly reduce body weight, with studies reporting average weight loss of around 3–4% of baseline weight over 12–15 months (96). However, compared to newer agents such as phentermine/topiramate and liraglutide, orlistat’s weight loss efficacy is less pronounced, and its impact on OSA may be correspondingly limited. In a real-world cohort, orlistat achieved a mean weight loss of 3.9% at 15 months, but patients with comorbid OSA were less likely to achieve clinically significant weight reduction, suggesting that the improvement in OSA severity may be suboptimal when using orlistat alone (96).

The therapeutic benefit of orlistat in OSA is likely mediated through weight loss, as reductions in adiposity can alleviate upper airway collapsibility and improve respiratory mechanics (116). Nevertheless, the magnitude of improvement in OSA parameters such as the AHI is generally proportional to the degree of weight loss, and orlistat’s modest efficacy may limit its standalone utility for OSA management. Additionally, orlistat is associated with gastrointestinal side effects, including steatorrhea, flatulence, and fecal urgency, which can lead to poor adherence and discontinuation. Another important consideration is the potential for fat-soluble vitamin deficiencies (A, D, E, K), necessitating regular monitoring and supplementation as appropriate (116). While orlistat remains a viable option for some patients, the emergence of more effective pharmacotherapies and the need for individualized treatment plans underscore the importance of weighing efficacy, tolerability, and patient preferences when selecting anti-obesity medications for the management of OSA and related comorbidities (117).

#### 2.3.3 Novel pharmacotherapies (e.g., phentermine/topiramate, zonisamide)

The combination of phentermine and topiramate has emerged as a promising pharmacological intervention for weight reduction in individuals with obesity and comorbid OSA. Phentermine/topiramate extended-release (ER) is a fixed-dose combination designed to decrease appetite and increase satiety, offering a dual mechanism for effective weight management. Clinical data indicate that this combination can yield clinically significant weight loss, with studies reporting a mean weight reduction of approximately 6.3% over 15 months in real-world settings, which is comparable to other leading anti-obesity medications such as liraglutide (96). Notably, the combination therapy has demonstrated efficacy in diverse populations, including both adults and adolescents, and has recently received approval for pediatric use in the United States for chronic weight management in those aged 12 years and older with severe obesity (118). Case reports further support the effectiveness of phentermine/topiramate in individuals with syndromic obesity, such as Bardet-Biedl syndrome, where substantial reductions in BMI and improvements in metabolic parameters have been observed (119). Importantly, weight loss achieved through pharmacotherapy, including phentermine/topiramate, has been shown to produce proportional improvements in OSA severity, as measured by reductions in the AHI (45). This relationship underscores the therapeutic potential of anti-obesity medications in addressing both weight and OSA-related outcomes.

In addition to phentermine/topiramate, zonisamide—a carbonic anhydrase inhibitor with established weight loss effects—has garnered attention for its potential dual role in OSA management. Beyond facilitating weight reduction, zonisamide’s mechanism of carbonic anhydrase inhibition may directly ameliorate OSA by modulating ventilatory control and reducing upper airway collapsibility. Although direct clinical trials of zonisamide in OSA populations are limited, its pharmacological profile and observed benefits in weight reduction suggest potential utility in this context (116). The evolving landscape of anti-obesity pharmacotherapy, exemplified by agents like phentermine/topiramate and zonisamide, reflects a paradigm shift toward integrated management of obesity and its comorbidities, including OSA. Ongoing clinical development and further research are warranted to delineate the long-term efficacy, safety, and direct effects of these novel agents on OSA outcomes (120).

#### 2.3.4 Limitations and safety of pharmacological therapy

Pharmacological interventions for weight loss in the context of OSA have gained increasing attention, particularly with the emergence of incretin-based therapies such as GLP-1 RAs and dual agonists like tirzepatide. While these agents offer promising dual benefits—promoting significant weight loss and improving OSA severity—they are not without limitations and safety concerns. The most commonly reported adverse effects of GLP-1 RAs and tirzepatide are gastrointestinal in nature, including nausea, vomiting, and diarrhea, which are generally mild and transient but can impact adherence and quality of life (121). Long-term safety data are still evolving, but current evidence suggests that these medications are generally well tolerated, with serious adverse events being rare (122). However, uncertainties remain regarding their effects over several years, particularly in populations with multiple comorbidities or those taking concomitant medications for chronic diseases such as cardiovascular disease or diabetes.

Patient selection is another critical limitation. Most studies have focused on individuals with obesity-related OSA, and the efficacy and safety profiles in non-obese patients or those with less severe OSA remain unclear (123, 124). Additionally, the interaction of anti-obesity pharmacotherapy with other chronic disease medications is a relevant concern, as many OSA patients have overlapping conditions requiring polypharmacy. Furthermore, contraindications exist for certain populations, such as those with a history of medullary thyroid carcinoma or pancreatitis, limiting the universal applicability of these drugs (121).

Finally, while pharmacological therapy provides a non-invasive alternative for those unable or unwilling to undergo bariatric surgery, its long-term sustainability and impact on cardiovascular outcomes in OSA patients are still under investigation (125, 126). The need for ongoing lifestyle modification alongside drug therapy remains paramount, as weight regain after discontinuation is a recognized challenge. In summary, while pharmacological therapy for weight loss in OSA represents a significant advancement, its use must be carefully individualized, considering potential side effects, long-term safety, patient comorbidities, and drug interactions, and should ideally be integrated within a multidisciplinary management framework.

### 2.4 Multiple mechanisms of weight loss on OSA

#### 2.4.1 Improvement of upper airway anatomy

Weight reduction plays a pivotal role in improving the anatomical structure of the upper airway, which is central to the pathophysiology of OSA. Obesity is recognized as the primary risk factor for OSA, primarily due to the accumulation of adipose tissue in the pharyngeal region, including the tongue and lateral pharyngeal walls. This fat deposition narrows the upper airway, increases collapsibility, and elevates the risk of obstruction during sleep. Recent studies employing magnetic resonance imaging (MRI) have provided direct evidence that weight loss, achieved through intensive lifestyle interventions or bariatric surgery, leads to significant reductions in tongue fat and soft tissue volumes surrounding the upper airway. Notably, the decrease in tongue fat volume has been shown to be the principal mediator of improved AHI, underscoring its critical role in OSA severity modulation. The correlation between reductions in tongue fat and improvements in AHI remains robust even after adjusting for overall weight loss, highlighting the specific impact of upper airway fat reduction on airway patency (60). Furthermore, bariatric surgery, as a potent weight loss intervention in obese patients, has demonstrated a marked decrease in upper airway adiposity, neck circumference, and palatal webbing, all of which contribute to enhanced upper airway patency and reduced risk of airway collapse during sleep (127). Pharmacological agents such as GLP-1 RAs, which promote substantial and sustained weight loss, may also mitigate OSA by reducing fat deposition around the upper airway, thereby decreasing soft tissue encroachment and airway resistance (111). Collectively, these findings affirm that weight reduction, regardless of the modality—surgical, pharmacological, or behavioral—directly translates into anatomical improvements of the upper airway, diminishing the propensity for airway obstruction and ultimately alleviating OSA severity.

#### 2.4.2 Improvement of metabolic and inflammatory status

Weight loss achieved through various interventions—including lifestyle modification, pharmacotherapy, and bariatric surgery—has demonstrated significant benefits in improving metabolic and inflammatory profiles, which in turn can mitigate the severity of OSA. Weight reduction is closely linked to enhanced insulin sensitivity and a marked decrease in systemic inflammation, both of which are key contributors to the pathophysiology of OSA. For example, a randomized clinical trial comparing lifestyle modification with CPAP therapy in obese patients with moderate to severe OSA found that lifestyle-induced weight loss led to greater reductions in high-sensitivity C-reactive protein (hsCRP), a marker of systemic inflammation, and improved insulin sensitivity compared to CPAP alone. Importantly, these metabolic improvements were observed regardless of craniofacial phenotype, underscoring the primary role of weight reduction in ameliorating OSA-related metabolic disturbances (128). The pro-inflammatory state associated with obesity exacerbates OSA through mechanisms such as endothelial dysfunction and neurohormonal dysregulation, which are alleviated as adiposity decreases (129, 130). Furthermore, anti-obesity pharmacotherapies—including GLP-1 RAs—have shown promise not only in facilitating substantial and sustained weight loss but also in reducing systemic inflammation and improving comorbid metabolic conditions, such as insulin resistance and hypertension, which are prevalent in OSA populations (111, 120). Bariatric and metabolic surgeries also contribute to long-term improvements in glycemic control, remission of type 2 diabetes, and reduction in inflammatory markers, thereby lowering the risk of microvascular and macrovascular complications associated with OSA (131). Collectively, these findings highlight the central role of weight loss in improving metabolic and inflammatory status, which indirectly alleviates OSA severity and enhances overall cardiometabolic health.

#### 2.4.3 Neuromodulation and autonomic function changes

Weight reduction has been shown to play a crucial role in modulating autonomic nervous system activity, particularly by decreasing sympathetic nervous system overactivity, which is a known contributor to increased cardiovascular risk and nocturnal blood pressure variability in patients with sleep apnea. The interplay between body weight and autonomic function is multifaceted, involving alterations in inflammatory mediators and neuroregulatory pathways. While the referenced study primarily investigates the neuroprotective effects of apelin-13 in a rat model of spinal cord injury, its findings underscore the broader concept that interventions targeting neural inflammation and promoting neuroregeneration can significantly enhance autonomic and functional recovery. Specifically, apelin-13 administration post-injury resulted in improved behavioral outcomes and reduced central nervous system inflammation, as evidenced by decreased pro-inflammatory cytokines and increased anti-inflammatory markers such as IL-10. These neuroprotective and anti-inflammatory effects contributed to better preservation of neural tissue and functional restoration, which are essential for optimal autonomic regulation (132). Translating these findings to the context of weight loss and sleep apnea, it is plausible that similar mechanisms—namely, the reduction of systemic and neural inflammation and the restoration of autonomic balance—underlie the observed cardiovascular benefits and stabilization of nocturnal blood pressure following weight reduction. Therefore, therapies that induce weight loss, whether through surgical, pharmacological, or lifestyle interventions, may confer their beneficial effects on sleep apnea not only by reducing upper airway collapsibility but also by improving autonomic regulation and mitigating cardiovascular risk through decreased sympathetic activity and enhanced neuroprotection.

### 2.5 Comparison and selection of different weight loss methods for the treatment of OSA

#### 2.5.1 Efficacy comparison

Bariatric surgery stands out as the most effective intervention for achieving substantial weight loss and improving OSA, particularly in patients with severe obesity and severe OSA. Multiple studies and meta-analyses demonstrate that surgical procedures such as RYGB, SG, and single-anastomosis duodeno-ileostomy with sleeve gastrectomy (SADI-S) lead to significant reductions in BMI and AHI, as well as high rates of remission for OSA and other obesity-related comorbidities (64, 133, 134). For instance, RYGB and SG have shown sustained weight loss and durable improvement in OSA over 5 to 10 years, with remission rates of OSA as high as 64.5% after RYGB and 50.1% after SG at 5 years (134, 135). SADI-S, both as a primary and revisional surgery, achieves excess weight loss rates exceeding 70% and OSA remission rates above 60% in the mid- and long-term (133, 136). Surgery is particularly recommended for patients with BMI ≥ 40 kg/m^2^ or ≥35 kg/m^2^ with serious comorbidities, where non-surgical approaches often fail to achieve adequate weight reduction or OSA control (64). In contrast, pharmacological therapies, especially the newer incretin-based agents such as GLP-1 RAs and dual GIP/GLP-1 agonists, have shown promising results in moderate weight loss (up to 20% of body weight) and corresponding reductions in AHI, offering a viable alternative for patients with mild to moderate obesity or those with contraindications to surgery (121, 122, 125). Anti-obesity medications like liraglutide and tirzepatide not only induce significant weight loss but also improve OSA severity and cardiometabolic risk factors, though the magnitude of AHI reduction is generally proportional to the degree of weight loss and may not match the results seen with surgery in severe cases (45, 120). Lifestyle interventions and structured weight management programs remain the cornerstone for patients with mild to moderate obesity and OSA, or as an adjunct to other therapies, but their efficacy is often limited by poor long-term adherence and modest weight loss, making them less suitable as sole therapy for severe cases (32, 79). Endoscopic procedures like endoscopic sleeve gastroplasty (ESG) may offer intermediate efficacy and are considered for select patients who are not candidates for surgery (137, 138). In summary, bariatric surgery delivers the most pronounced and sustained improvements in both weight and OSA severity, especially in patients with severe obesity and OSA, while pharmacotherapy and lifestyle interventions are appropriate for those with mild to moderate disease or when surgery is contraindicated, underscoring the need for individualized treatment selection (45, 64, 122).

#### 2.5.2 Individualized treatment strategies

Developing individualized treatment strategies for weight reduction and OSA management hinges on a comprehensive assessment of each patient’s obesity severity, comorbidities, adherence potential, and risk preferences. The heterogeneity in OSA pathophysiology and its close association with obesity necessitate a multifaceted and tailored approach. For instance, the severity of obesity and the presence of comorbidities such as cardiovascular disease, diabetes, or hypertension significantly influence both the urgency and the selection of therapeutic modalities. The Obesity Medicine Association highlights the importance of early and sustained intervention, recommending a combination of nutrition therapy, physical activity, behavioral counseling, pharmacotherapy, and bariatric procedures, with the choice and frequency of interventions personalized to maximize outcomes and preserve lean body mass (89). Similarly, the European Association for the Study of Obesity (EASO) framework emphasizes the need for evidence-based, individualized pharmacological interventions, taking into account patient-specific factors such as BMI, comorbidities, and treatment response to optimize both efficacy and safety (117).

In OSA management, CPAP remains the cornerstone for moderate to severe cases, but adherence is often suboptimal, prompting the need for alternative or adjunct therapies. Patient characteristics—including anatomical sites of airway obstruction, identified through modalities such as drug-induced sleep endoscopy (DISE), and physiological traits—should guide the selection of therapies such as oral appliances, upper airway surgeries, or emerging pharmacological agents like GLP-1 RAs and dual incretin therapies, which not only promote weight loss but also improve OSA severity (123, 139). Behavioral interventions, including structured weight management programs and self-monitoring, are crucial for long-term success and must be tailored to individual lifestyle, motivation, and support systems. Furthermore, patient preferences and risk tolerance—whether for surgical intervention, medication side effects, or intensive lifestyle modification—must be integrated into shared decision-making to enhance adherence and satisfaction (140, 141).

Ultimately, the paradigm of personalized medicine in obesity and OSA care is evolving to incorporate not only clinical and physiological parameters but also patient-reported outcomes, quality of life, and psychosocial factors. The integration of multidisciplinary teams—including sleep specialists, bariatric surgeons, nutritionists, and behavioral therapists—ensures that individualized treatment plans are comprehensive, dynamic, and responsive to the changing needs of patients across the spectrum of obesity and OSA severity (139, 142). This approach maximizes the likelihood of sustained weight reduction, improved OSA control, and long-term health benefits.

#### 2.5.3 Combined treatment models

The combined application of multiple weight loss strategies—such as pharmacotherapy with behavioral interventions, or surgery alongside lifestyle management—has emerged as a highly effective approach in the management of obesity and its related comorbidities, including OSA. Integrative medicine, which coordinates conventional therapies like CPAP and oral appliances with weight loss and lifestyle programs, has demonstrated improved symptomatic outcomes and a reduction in the AHI in OSA patients (143, 144). Recent advances in pharmacological therapies, particularly with incretin-based agents such as GLP-1 RAs, have shown significant potential not only for weight reduction but also for improving metabolic and respiratory parameters in OSA (108, 145, 146). These agents, when used in combination with lifestyle interventions or as adjuncts to surgical procedures, can address the multifactorial nature of obesity and OSA, potentially leading to greater and more sustainable reductions in disease severity. Evidence also suggests that bariatric and metabolic endoscopic therapies, when paired with lifestyle changes, offer a safer and effective alternative to traditional surgery, achieving clinically meaningful weight loss and improvement in OSA symptoms (147). Moreover, emerging combination therapies—such as dual or triple hormonal receptor agonists—are being developed to maximize weight loss and optimize tolerability, further enhancing the prospects of long-term disease control (120). Importantly, multidisciplinary and personalized strategies, which integrate behavioral, pharmacological, and surgical modalities, have been shown to lower relapse rates and improve adherence, particularly in complex cases where single-modality interventions are insufficient (148). As the landscape of obesity and OSA management evolves, a comprehensive, individualized approach that leverages the synergistic effects of combined therapies is increasingly recognized as the optimal pathway to achieving sustained weight reduction and mitigating OSA-related health risks. It is vital to emphasize again the importance of developing novel patient treatment protocols and methods, such as integrating digital health tools and personalized behavioral strategies, to enhance adherence and efficacy in OSA management.

### 2.6 Challenges and future directions in long-term management of OSA with weight loss

#### 2.6.1 Long-term adherence and weight maintenance

Achieving and sustaining long-term weight loss is a central challenge in the management of OSA, particularly given the high risk of weight regain and the critical importance of ongoing adherence to therapeutic strategies. Multiple factors influence long-term adherence, including biological, psychological, behavioral, and environmental components. Behavioral weight loss programs, while effective in the short term, often see diminished adherence over time, leading to weight regain; this is supported by evidence that dietary adherence, rather than specific macronutrient composition, is the primary predictor of long-term weight loss success (149). Psychological factors such as impulsivity, emotional eating, and depression have also been identified as significant predictors of weight maintenance after interventions like bariatric surgery, highlighting the need for comprehensive pre- and post-surgical psychological evaluation and ongoing support (150, 151). Cognitive training to strengthen executive functions—such as self-regulation and self-efficacy—may further enhance adherence and weight maintenance, although more research is needed to establish its efficacy (152). Additionally, regular self-monitoring behaviors, such as dietary tracking and frequent self-weighing, are consistently associated with better weight maintenance, with evidence suggesting that self-monitoring at least 3 days per week, and ideally 5 to 6 days, yields the greatest benefit (153). The integration of technology, including telemedicine and eHealth interventions, provides innovative platforms for delivering ongoing support, personalized feedback, and behavioral reinforcement, which can mitigate barriers related to time, access, and motivation and have been shown to improve long-term adherence and weight loss maintenance (154, 155). Pharmacotherapy, when used in conjunction with behavioral interventions, is recommended for sustained weight loss and maintenance, and long-term use of approved anti-obesity medications has been shown to facilitate clinically meaningful weight loss and improve OSA-related health outcomes, though adherence to pharmacotherapy itself must also be monitored (39, 156, 157). Bariatric surgery remains the most effective intervention for severe obesity, but sustained success depends on adherence to dietary and lifestyle changes postoperatively, with comprehensive follow-up programs and tailored interventions playing a pivotal role in preventing weight regain (151, 158). Ultimately, a multifaceted, personalized approach—incorporating behavioral, psychological, technological, and pharmacological strategies, along with ongoing professional support—is essential to enhance long-term adherence and prevent weight rebound in OSA management.

#### 2.6.2 Prospects for novel pharmacological agents and surgical techniques

The landscape of OSA management is rapidly evolving with the advent of novel pharmacological agents and minimally invasive surgical techniques, offering new hope for patients who are unable to achieve adequate control with traditional therapies. Among the most promising developments is the emergence of new-generation GLP-1 RAs and dual agonists such as tirzepatide, which have demonstrated significant efficacy in promoting weight loss and improving OSA severity. The recent FDA approval of tirzepatide for the management of obesity in adults with OSA underscores its dual benefit in targeting both metabolic dysfunction and sleep-disordered breathing, marking a significant milestone in personalized OSA therapy (123, 124). These incretin-based therapies not only facilitate substantial weight reduction but also appear to directly ameliorate OSA symptoms, likely by mitigating the underlying metabolic and inflammatory drivers of upper airway collapsibility. Furthermore, emerging evidence suggests that combination pharmacotherapy and agents targeting lipid metabolism, such as cholesteryl ester transfer protein (CETP) inhibitors, may also play a role in reducing OSA risk, highlighting the potential for multi-modal drug strategies in the future (159). On the surgical front, the refinement of minimally invasive procedures—such as ESG—provides a viable alternative for patients with severe obesity who are unsuitable for conventional bariatric surgery. ESG has been shown to achieve meaningful weight loss and facilitate discontinuation of CPAP therapy in select patients, with a favorable safety profile and rapid recovery (138). Additionally, advances in diagnostic modalities like DISE enable more precise identification of airway obstruction sites, facilitating tailored surgical interventions with potentially greater efficacy and fewer complications (139, 160). Collectively, these innovations herald a future in which OSA management is increasingly individualized, integrating pharmacological advances, combination therapies, and minimally invasive surgical options to optimize patient outcomes.

#### 2.6.3 Multidisciplinary collaboration and integrated management

The management of OSA, particularly in the context of obesity and weight reduction, increasingly relies on a multidisciplinary collaboration that spans sleep medicine, nutrition, endocrinology, surgery, dentistry, and other allied health disciplines. This integrated approach is essential due to the multifactorial etiology and complex comorbidity profiles commonly seen in OSA patients. Evidence from both clinical practice and research strongly supports the value of multidisciplinary teams (MDTs) in optimizing diagnosis, tailoring treatment plans, and improving outcomes for patients with OSA. For example, the involvement of sleep medicine specialists ensures accurate diagnosis and ongoing assessment, while nutritionists and endocrinologists address metabolic risk factors and guide sustainable weight management strategies. Surgeons provide expertise in bariatric or upper airway surgical interventions when indicated, and dentists or orthodontists contribute to screening, early detection, and management through oral appliance therapy or craniofacial interventions, highlighting the necessity for standardized protocols and shared pathways to care (161, 162). Clinical guidelines and consensus statements emphasize continuous, collaborative follow-up to monitor treatment efficacy, reduce recurrence risk, and address evolving patient needs (163, 164). In pediatric populations, multidisciplinary collaboration is especially critical, as early intervention involving pediatricians, ENT specialists, orthodontists, and sleep experts can prevent long-term complications and improve quality of life (165, 166). Furthermore, successful case reports and retrospective analyses demonstrate that MDTs can effectively coordinate preoperative optimization, perioperative management, and postoperative care, leading to safer surgical outcomes and better long-term weight control in severely obese patients with OSA (167, 168). The inclusion of behavioral health professionals, physical therapists, and patient educators further supports adherence to therapy, lifestyle modification, and psychosocial well-being (169, 170). Overall, the literature consistently underscores that multidisciplinary collaboration is not only beneficial but indispensable for comprehensive, patient-centered OSA management, ensuring that interventions are personalized, evidence-based, and responsive to the full spectrum of patient needs.

## 3 Conclusion

Obesity and OSA are closely interrelated, with excess body weight serving as a major modifiable risk factor for OSA onset and progression. Within this context, weight reduction has emerged as a fundamental pillar in OSA management, supported by robust evidence for its efficacy across the spectrum of disease severity. However, the landscape of weight loss interventions is diverse, with each approach offering distinct advantages, limitations, and implications for patient care. As experts in the field, it is crucial to critically assess and balance these modalities in pursuit of optimal, individualized patient outcomes.

Bariatric surgery remains the most effective intervention for substantial and sustained weight loss, particularly in patients with severe obesity and OSA. Numerous studies have demonstrated marked improvements in OSA severity post-surgery, often leading to significant reductions in AHI and, in some cases, disease remission. However, surgical interventions require careful patient selection, comprehensive perioperative assessment, and long-term follow-up to manage potential complications and address the risk of weight regain. The invasive nature of surgery, its cost, and the need for lifelong nutritional and medical monitoring necessitate prudent weighing of benefits versus risks, especially in populations with comorbidities or limited access to specialized care.

Pharmacological treatments, particularly the advent of novel agents such as GLP-1 RAs, have expanded therapeutic options for patients unable or unwilling to undergo surgery, as well as those with mild to moderate obesity. These medications have shown promising results in achieving clinically meaningful weight loss, with emerging evidence supporting their role in OSA improvement. Nonetheless, long-term efficacy, safety, and accessibility remain areas for ongoing investigation. The potential for adverse effects, variable patient responses, and the need for sustained therapy highlight the importance of integrating pharmacotherapy within a broader, multidisciplinary management framework.

Conventional lifestyle interventions, including dietary modification, increased physical activity, and behavioral therapy, are foundational strategies suitable for a wide range of OSA patients. While the magnitude of weight loss achieved through these measures is typically modest compared to surgical or pharmacological approaches, their non-invasive nature and applicability to diverse populations make them indispensable. However, their effectiveness is heavily contingent upon patient adherence and the availability of structured support systems. High dropout rates and weight regain are common challenges, underscoring the necessity for ongoing patient engagement, education, and personalized goal-setting.

Mechanistically, weight loss alleviates OSA not only by reducing pharyngeal fat deposition and improving upper airway patency, but also by ameliorating systemic metabolic dysfunction and modulating neurohumoral regulation. These multifaceted pathways highlight the interconnectedness of obesity, OSA, and related comorbidities, reinforcing the value of comprehensive weight management to improve both respiratory and overall health outcomes.

From an expert perspective, the optimal management of OSA in obese patients must be individualized, multidisciplinary, and longitudinal. Integrating the expertise of sleep medicine specialists, bariatric surgeons, endocrinologists, nutritionists, and behavioral therapists is essential for tailoring interventions to patient preferences, comorbidity profiles, and readiness for change. Individualized treatment plans should consider not only the efficacy and risks of each weight loss modality, but also patient adherence, psychosocial factors, and long-term sustainability.

While AASM guidelines position surgical interventions as a last resort for primary OSA treatment due to risks and inconsistent outcomes, bariatric surgery uniquely reduces OSA risk in obese patients by addressing underlying obesity, yielding significant AHI reductions (e.g., −20 events/h) and remission rates up to 70% through weight loss and metabolic remodeling (Section 2.1.2). This must be contextualized as an obesity-targeted approach rather than direct OSA surgery, reserved for severe cases (BMI ≥35 kg/m^2^ with comorbidities) where it surpasses non-surgical durability, balanced against perioperative complications (Section 2.1.4). Integrating it into personalized, multidisciplinary frameworks reconciles this with guidelines (41).

Looking forward, research should focus on refining the integration of emerging weight loss therapies within OSA management algorithms, identifying biomarkers to predict treatment response, and developing strategies to enhance adherence and minimize relapse. Large-scale, high-quality studies are needed to compare the long-term outcomes of different interventions, including their impact on OSA-related morbidity, cardiovascular risk, and quality of life. There is a critical need for advancing OSA diagnosis and treatment methods, including enhanced polysomnography with biomarkers, sex-tailored behavioral interventions to mitigate underdiagnosis (e.g., in women), and novel multidisciplinary protocols for comorbidities like sleep bruxism, incorporating lifestyle, sleep hygiene, diet, and psychosocial elements such as intimacy to improve sleep continuity and reduce stress (171, 172). These developments could improve precision, accessibility, and patient outcomes, filling current gaps.

In summary, weight reduction remains a cornerstone of OSA management, with surgical, pharmacological, and lifestyle interventions each playing critical but distinct roles. Achieving sustained improvements in OSA outcomes requires a patient-centered, multidisciplinary approach that balances efficacy, safety, and long-term adherence. Advancing our understanding of the complex interplay between weight loss and OSA pathophysiology will enable more precise, effective, and durable treatment strategies, ultimately improving the prognosis and quality of life for this growing patient population.
